# Medical Students and the War

**Published:** 1915-01-09

**Authors:** 


### The Hospital

The Workers' Newspaper of Administrative Medicine and Institutional Life, Administration, National Insurance and Health.

No. 1490, Vol. LVII. SATURDAY, JANUARY 9, 1915. PRICE ONE PENNY.

### MEDICAL STUDENTS AND THE WAR.

The probable effect of the war on the supply of medical practitioners is now taking a definite and readily estimated form. The figures show that students from all stages of the curriculum have in large numbers abandoned their studies, in order, doubtless, to render help in some form or other to the fighting Services. From the Edinburgh School alone, according to Principal Sir Wm. Turner, some hundreds of undergraduates have given up their academic position for a military career. More comprehensive, and still more precise, are the figures published on the authority of the President of the General Medical Council. From these it appears that the decrease in enrolments in 1914, as compared with the figures of the previous year, is: First year 56, second year 237, third year 237, fourth year 211, fifth (and higher) year 300; that is, in round numbers, there are about 1,000 medical students less to-day than there were in 1913. When with this deficiency of supply there is associated the increased deaths of practitioners due directly or indirectly to the war, it is obvious that there is shortly to be at the service of the public a smaller number of practitioners than has hitherto been the case. Unless the present supply of doctors is excessive—and at the highest we think this is true only in a limited sense—the prospect for the community is not without anxiety.

As up to the present doctors have for the most part been readily obtained, as they are paid fees for their services, and as they have generally shown themselves quite ready to work without being paid, there is a risk of their public values being overlooked. Yet a definite deficiency in the supply of medical practitioners in any neighbourhood would be a very serious matter for the inhabitants. In addition to defective relief of the patient, there is the alarm and anxiety of friends, who are quite unable to distinguish between what is trifling and what is serious, and who, in their desire to "do something," may easily do harm. The same principle applies also to less acute situations. In short, a sufficient number of medical practitioners is an essential public interest, and necessary both for the bodily safety and for the peace of mind of our citizens. The present situation certainly lends force to this contention. It also affords a justification for those who, in various agitations in recent years, have advocated policies calculated to make more useful and attractive the career of the general practitioner. There have, as our readers will recollect, been public proposals, partly of lay origin, partly fostered within the profession itself, which, if adopted, would have meant the displacement of the local practitioner in favour of an imported, junior, so-called "specialist." Protests against these proposals have been characterised and denounced as professional selfishness. Is it not necessary, it has been asked, that poor people, or young children, or whatever may be the immediate philanthropic object, should be provided with the best and most skilful medical advice and assistance? Such a suggestion obviously begs the question what is the best medical advice and assistance, and, still more, it neglects the interest now brought prominently to the front by the impending diminution in the supply of medical practitioners. If public policy is so directed that it makes general practice devoid of interest and opportunity, the supply of general practitioners will most certainly diminish, and the public evils and inconveniences which must result from such diminution will inevitably appear. Hence, we repeat, whatever tends to increase the attractions of general practice may be advocated as resting upon the sound basis of public interest and not upon the narrow ground of selfish and class interest. Thus we come back to our original proposition that the figures showing a very decided decrease in the students of medicine is a matter for public concern.

The mitigation of the evil can be secured only by increasing the numbers of medical students. Towards this end the publicity of the facts may do something, as it will turn the thoughts of youths and their parents to the prospects offered by a medical career. A lad of sixteen or seventeen years, though too young for the Army, may quite well commence the study of medicine. Of great importance, too, is the advice issued by the President of the General Medical Council—namely, that students who have made some substantial progress with their studies should at least complete the curriculum, and should graduate before turning their steps towards the Army. The welfare of the community demands doctors as well as soldiers, and the answer to the one claim is not less patriotic than a response to the other. Already our hospitals and similar institutions have difficulty in securing junior members for their staffs, and this cannot fail to act to the prejudice of patients. The extension of a similar deficiency to the community generally would be a very serious evil.

